# The Imaginary Part of Coherency in Autism: Differences in Cortical Functional Connectivity in Preschool Children

**DOI:** 10.1371/journal.pone.0075941

**Published:** 2013-10-01

**Authors:** Luis García Domínguez, Jim Stieben, José Luis Pérez Velázquez, Stuart Shanker

**Affiliations:** 1 Temerty Centre for Therapeutic Brain Intervention, Centre for Addiction and Mental Health, University of Toronto, Toronto, Ontario, Canada; 2 Milton and Ethel Harris Research Initiative, York University, Toronto, Ontario, Canada; 3 Neuroscience and Mental Health Programme, Brain and Behaviour Centre, Division of Neurology, Hospital for Sick Children; Department of Paediatrics and Institute of Medical Science, University of Toronto, Toronto, Ontario, Canada; Bellvitge Biomedical Research Institute-IDIBELL, Spain

## Abstract

Cognition arises from the transient integration and segregation of activity across functionally distinct brain areas. Autism Spectrum Disorders (ASD), which encompass a wide range of developmental disabilities, have been presumed to be associated with a problem in cortical and sub-cortical dynamics of coordinated activity, often involving enhanced local but decreased long range coordination over areas of integration. In this paper we challenge this idea by presenting results from a relatively large population of ASD children and age-matched controls during a face-processing task. Over most of the explored domain, children with ASD exhibited enhanced synchronization, although finer detail reveals specific enhancement/reduction of synchrony depending on time, frequency and brain site. Our results are derived from the use of the imaginary part of coherency, a measure which is not susceptible to volume conduction artifacts and therefore presents a credible picture of coordinated brain activity. We also present evidence that this measure is a good candidate to provide features in building a classifier to be used as a potential biomarker for autism.

## Introduction

A significant challenge in the study of autism is to understand how the integration of brain activity occurring at multiple levels – cells and networks – results in the behaviors that are highly characteristic of the disorder. While advances have been made in our understanding of the genetic bases of autism and considerable effort has focused on neuroimaging the brains of individuals with Autism Spectrum Disorders (ASD), very little is known about the dynamics of the brains of individuals with ASD. More specifically, if we could associate particular brain coordination dynamics with specific behaviors, this could result, not only in a basic understanding of how characteristic behaviors of ASD result from altered neurodynamics, but also in the development of specific biomarkers that could help in the early diagnosis of ASD. Furthermore, such a mapping would afford the possibility of applying targeted interventions designed to enhance the integration of brain activity in young children with ASD.

In this study, we used dense-array scalp electroencephalographic (EEG) recordings to identify distinctive patterns of coherency in children with autism during attention to faces as compared to age matched controls. The characteristic behaviors of children with ASD suggest that their brains may process information differently from their typically developing age-matched peers. Some theoretical models have been proposed to explain these differences. For example, according to the weak central coherence theory [1], individuals with autism tend to over focus on details and have difficulty integrating contextual information. This problem was later theorized to be caused by reduced integration between brain networks [2], and more recently interpreted in terms of reduced global and increased local connectivity/synchronization. Most results from fMRI studies seem to support the thesis that reduced intracortical connectivity results in a lower degree of integration of information across certain cortical areas [3], [4].

Few studies have addressed the problem of functional connectivity in autism from the perspective of electrophisiological recordings (EEG/MEG). In a recent study [5] coherence analysis was applied to spontaneous EEG from a large population of children with ASD and age matched controls, documenting reduced short-distance and increased long-distance coherences in ASD. However this study was limited to a reduced set of networks after pruning the data using a principal component analysis. Murias et al., 2007 [6] applied coherence to high density EEG from an adult population, finding robust patterns of over- and under-connectivity at distinct spatial and temporal scales in an eyes-closed resting state. In another study using resting state EEG in children, Coben et al (2008) [7], reported a general pattern of under-connectivity (coherence) as well as some over-connectivity over specific frequency bands and regions. Perez Velazquez et al 2009 [8] conducted a MEG study using phase synchronization over a sliding temporal window and idenitfied a decrease of connectivity but also some over-connectivity, specifically over parietal regions in subjects with ASD.

Modern theories of brain function propose that cognition is based on the integration of information derived from diverse modes of perception in different specialized brain areas. Information processing consists of the coordinated integration of transient activity between distinct brain regions. This integration is based on neural synchronization, a phenomenon by which different areas of the brain tune into each other at specific frequencies for short-lived periods of time [2], [9]–[11].

Understanding the essential mechanisms underlying functional connectivity in brain circuits is crucial to a proper comprehension of their role in adaptive and pathological processes. For this reason, coordinated activity in widespread brain areas is being studied in normal and pathological conditions. The disorders most investigated are epilepsy, movement disorders, and schizophrenia (reviewed in [12]).

A nonflat EEG can only arise from coordinated activity, in phase, of a local neuronal population. Traditional event-related potential analysis depends on this specific type of coordinated activity. However, to study the extension of this phenomenon – viz., long-range coordination -- one needs special tools since such coordinated activity is mainly manifested by delayed communication (non null phase difference introduced by the neuronal transmission time characteristics) at different frequency bands [13]. Thus, for the study of such phenomena, analysis methods of functional and effective connectivity in the frequency domain are more insightful.

One intensive area of research in neuroscience is the development of robust measures to characterize brain coordination dynamics from brainwaves. Many methodologies have been proposed so far, exploring different aspects of coordinated activity. These methodologies have different advantages and disadvantages especially when applied to EEG and MEG recordings. The problem of volume conduction, the superposition of many sources over each single sensor along with the presence of secondary currents, and the problem of EEG montage, militate against a straightforward interpretation when the analysis is performed on the sensor space [14]–[17].

Coherence is a measure that has been widely used to infer synchrony between different areas at the sensor level. The main weakness of coherence is that it is strongly affected by volume conduction. Recently new methods have been proposed which eliminate this problem. One of these new measures, the Imaginary Part of Coherency (ICOH), proposed by Nolte in 2004 [18], is aimed at eliminating all sources of extraneous coherence that are a consequence of instantaneous activity. What is left, the Imaginary Part, captures true source interactions at a given time lag. The method has a 100% positive predictive value, which means that whenever it produces significant values some coordinated activity is taking place. In the author’s words, “non-interacting sources cannot explain a nonvanishing imaginary coherency” [19].

While most of the conclusions regarding the functional connectivity observed in brain activity of individuals with ASD have been derived from metabolic measures such as PET or fMRI, data from electrophysiological recordings (e.g., EEG or MEG) are better suited to capture the transient and dynamic coordination between neural networks because of the combination of high temporal resolution and ability to conduct separate analyses at different frequency bands. Moreover, the ICOH, a promising tool for functional connectivity assessment, has not been used in the exploration of ASD and brings a new lens to a field dominated by neuroimaging, where most analyses are based on zero-lag correlation.

Our study provides a new perspective on the current debate regarding the "disconnected" autistic brain by assessing these imaginary coherency patterns in order to explore functional coordination of the brain in this group of children. We also propose that imaginary coherency can be potentially used diagnostically for the detection of phenotypes of autism early in development.

## Methods

This research has been reviewed and approved by the Human Participants Review Sub-Committee, York University’s Ethics Review Board and conforms to the standards of the Canadian Tri-Council Research Ethics guidelines.

Thirty-one typically developing (age ranges 2 to 5 years) and seventy-two children (age ranges 2 to 4 years 11 months) diagnosed with autism participated in the study. All children with ASD were previously diagnosed hovever, we confirmed the diagnosis using the following diagnostic instruments: ADI (Autism Diagnostic Inventory [20]) and the ADOS (Autism Diagnostic Observation Schedule [21]). Typically developing subjects were screened for a history of developmental, psychiatric or neurological disorders. Typically developing and autistic subjects were age matched. All parents signed consent forms prior to entry into the experiment.

Stimuli consisted of fifty randomly presented pictures of female faces displaying fearful and happy emotional expressions using photos acquired from models and mothers of participants in the study. Luminosity was controlled for all pictures. Pictures (3 by 5 inches) were presented using E-Prime and were randomly displayed with a duarion between 1200 to 1500ms and a 500ms ISI. A fixation point was presented prior to stimulus onset and was displayed randomly between 750 and 1200ms. Emotion and familiarity were not analyzed in the current study but were used in a larger randomized control treatment outcome study assessing treatment outcomes from therapy. Brain activity was monitored using 128 channel EGI geodesic electrode caps (Electrical Geodesics Inc., Eugene, USA). Children were trained for up to four weeks using a mock 128 channel electrode cap in order to desensitize them to the net. Children were also given up to six play sessions in the lab to get comfortable with the lab and testing equipment. During testing, children were seated in a comfortable chair and mothers were able to sit next to the subjects during testing. Eye-gaze activity was monitored using a Tobii eye-tracking camera (X50). Eye gaze activity was recorded for each trial in the EEG track and only those trials with fixation on the face for more than 100 ms beginning at the stimulus onset were used for analysis. Face stimuli were presented to participants until, at least, 50 trials with this gaze criteria were acquired per condition. In some cases we could afford more than that.

The data are contaminated with multiple artifacts including different eye-induced, electrode, head movement and EMG-induced artifacts. To deal with these sources of noise and the large amount of data in a reasonable time, a supervised machine-learning algorithm for automatic artifact rejection was designed. Initially a catalogue of artifacts and non-artifacts was obtained based on visual information from a number of trials within 20 subjects. The visual information consisted of the first 20 time courses from a Principal Component decomposition, along with their power spectrum and their projection over the scalp. In our experience this spatio-temporal information makes most artifacts easy to identify. Some components, examined within each trial, are then labeled as “good” or “bad” and stored in a database (the catalogue) along with a number of variables, or features, extracted from the corresponding component (standard deviation of the score, kurtosis of the score, power spectrum, loadings, kurtosis of the loadings, etc). Components that are not labeled are not stored. Those unlabeled components correspond to situations in which we were undecided. Thus, each entry in this catalogue corresponds to a component from a specific trial for a specific subject. Having obtained more than 5000 samples (of bad and good components), we used the database to train a classification tree. Once the optimal parameters of the tree are determined we used it to automatically remove components from every trial from every subject. No entire trial was removed. Only clearly artifactual components within each trial were eliminated by deleting the component and applying the inverse transformation. An evaluation of the performance of the classifier on randomly sampled trials from different subjects not in the catalogue showed an agreement of around 93% with the human classification. This is well within the margin of uncertainty about the nature of some components. In general we adopted the criteria of avoiding the removal of components whose nature is not very clear. Even so, some ambiguity is always present. The classifier showed a sensitivity of almost 100%, that is, every clear artifact is always removed from the data.

This cleaner dataset that was originally acquired in a reference montage using channel Cz was then converted to a reference free montage, using the Current Source Density (CSD) toolbox [22]. This technique was employed because previous results show that the calculation of coherence and synchronization measures on reference montages produces misleading results [15], [16], [23]. In the next step the eighteen marginal channels were removed from the original montage since these are more likely to contain a high percentage of EMG power [24]. This also alleviates the computational load of calculating coherency values for each pair of channels.

To determine the degree of synchronization between two brainwaves recorded at two specific sensors, the Imaginary Part of Coherency [18] was calculated using an adaptation of the EEGLAB function *newcrossf()* that computes the phase coherence (ERPCOH) [25] as an event-related activity. The adaptation consists in obtaining the imaginary part of the complex coherency number instead of its absolute value. 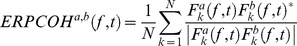



Here *N* is the number of trials and 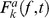 is the fourier coeficient for trial *k* at frequency *f* for a window centered at time *t* at channel *a*. The term 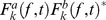 is the cross-spectrum between two given time series from *a* and *b* and is normalized by its absolute value in the formula. From this formula we are only interested in the absolute value of the Imaginary part of the resultant coherency vector (ICOH). The parameters supplied to the *newcrossf()* function were the ones already implemented by default.

The Fisher’s Z transformation was initially applied to the coherency values in order to help stabilize its variance [18], [26]. However the number of trials (N) for each subject was highly variable and we found the values of ICOH were strongly dependent on this parameter. Since we were interested mostly in the absolute value of the imaginary coherency we determined that these values were very influenced by N. While the average of the signed ICOH fluctuates around zero its absolute value depends linearly on the standard deviation. In order to remove these dependencies we followed an empirical approach. The standard deviation was found by regression to be consistent with the following model (x*N)^y^, where y was found to be –1/2 and x very close to 2. Thus, we applied the inverse transformation to each data point (that is, multiplying them by 2N^1/2^). ). Using this transformation the bias imposed by N was corrected, the variance was effectively uncorrelated to the number of trials and so the absolute value of the icoh. The exact value of x contributes only to produce a standard deviation equal to 1, but does not have any influence on the dependency over N. To simplify, in what follows, the term ICOH is used to denote the absolute value of ICOH.

To summarize, a value of ICOH was obtained for: each pair of channels (5995 pairs from 110 sensors), each of 26 frequency values, equally spaced from 2 to 55 Hz, each time point (200 samples from –470 ms to 870 ms after stimulus), and for each of the face categories for a total of 124, 696, 000 values per subject.

The total calculation across all subjects took approximately 4 days using Matlab (© 2011 The MathWorks, Inc.) capability of parallel processing (Parallel Computing Toolbox™) over between 16 to 32 cores distributed over SHARCNET, a consortium of Canadian academic institutions who share a network of high performance computers.

## Results

### Subject x Connection x Time x Frequency x Task

As explained earlier, all the results are derived from a single 5-dimensional data matrix of ICOH values, whose dimensions ([103 5995 200 26 4]) correspond to number of subjects, pair of channels, time points, frequencies, and face categories respectively. Of the 103 subjects, 31 belonged to the Control group and the remaining 72 to the ASD group. All data used for subsequent statistical analysis are obtained from this basic matrix by collapsing, averaging across some dimensions or segmentation in sub-matrices.

**Figure 1:**
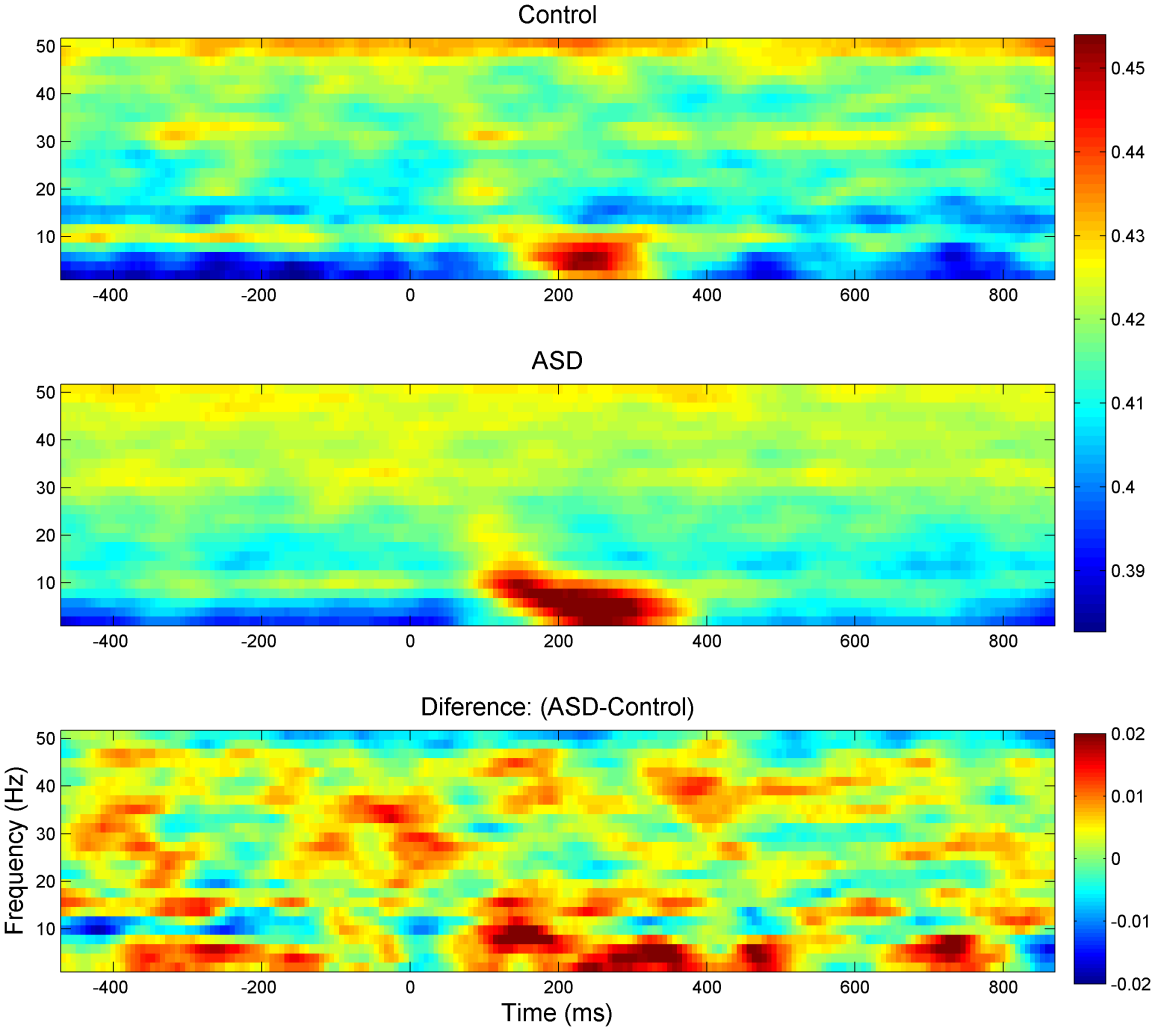
Time-Frequency plots of ICOH values corresponding to control group (top panel), ASD (middle panel) and their difference (bottom panel). Channels, Tasks and Participants have been pooled together and averaged within each group.

Gross differences in ICOH between the ASD and Control groups can readily be seen in a time-frequency plot (figure 1) where all ICOH values, for all channels and face categories, are averaged across participant in each group. As the time frequency plot shows, a characteristic pattern of higher ICOH values occurs during the post-stimulus time, specifically during the window 100–350ms, and particularly notable in the ASD group at lower frequencies, from 1 Hz to around 18 Hz, where there seems to be a progression from lower beta to lower frequencies. At higher frequencies there is also a generally enhanced ICOH for the ASD group but no clear pattern associated to the stimulus. During the pre-stimulus period around 10 Hz there seems to be lower synchronization in the ASD group.

**Figure 2:**
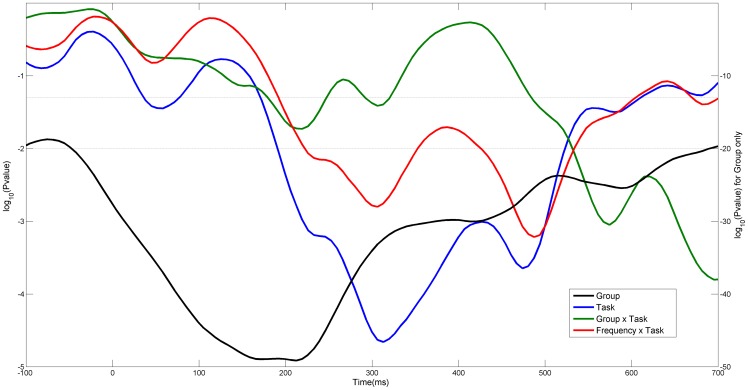
Curves are base-10 logarithms of Pvalues, from a number of 3-way ANOVA’s. ANOVA’s were repeated for 181 sliding time windows. Within each single time window 20 ICOH values were averaged corresponding to a period of 128ms. The 3 factors in the analysis were, the two Groups, Frequencies (considered here as a continuous factor) and Task. The y-axis on the left correspond to factors Task, Group x Task and Frequency x Task. The one on the right is only for Group.

In order to test the effects of the factors “Task” and “Frequency” in their interaction with the data from the ASD and control groups, we performed a number of 3-way analyses of variance (ANOVA), each for a different time window. ICOH values were averaged over a sliding time window in order to pinpoint variations in the p-value in relation to the presentation of the stimulus. This produced 181 ANOVA’s. Figure 2 depicts the p-values for the terms Group, Task, and the interactions Frequency x Task and Group x Task. The two dashed lines stand for the two common significant alpha levels 0.05 and 0.01. The y-axis to the left correspond to the Group factor only.

The p-value of the term Group is significant across all time points but reaches its lower value around 200 ms. Task becomes significant around 200 ms after the stimulus and reaches bottom around 300 ms. The interaction terms also become significant around 200 ms but their significance is not as solid as with the single terms. The terms Frequency and Group x Frequency are not shown since their p-values are even lower than those of Group across all time points.

### Subject x Connection x Time x Frequency

Since the highest statistical significance is found for the factors Group and Frequency, and their interaction, we decided to focus on the general differences of face processing, collapsing the four conditions or tasks into a single one. The analysis of the details of the differential response to task will be the subject of a future study. To produce a reliable estimation of single ICOH values derived from the entire collection of face conditions, the estimation of such values for the four tasks was added after weighting them by the number of trials of each condition.

**Figure 3:**
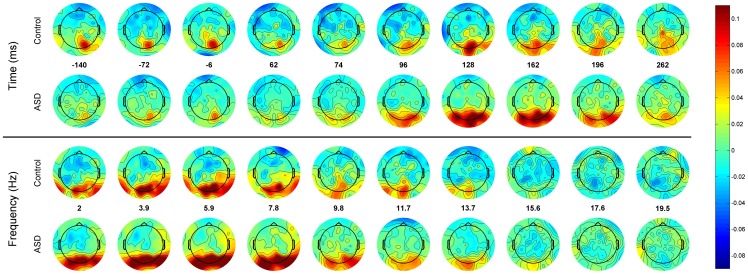
. Topographic view of ICOH values. The average of ICOH values of a single channel is mapped to the position of the channel. Values are interpolated for areas between electrodes. Top panel displays the average head of each group for different time points at a fixed frequency (9.8 Hz). Bottom panel the frequency is then varied and the time fixed (196ms).

**Figure 4:**
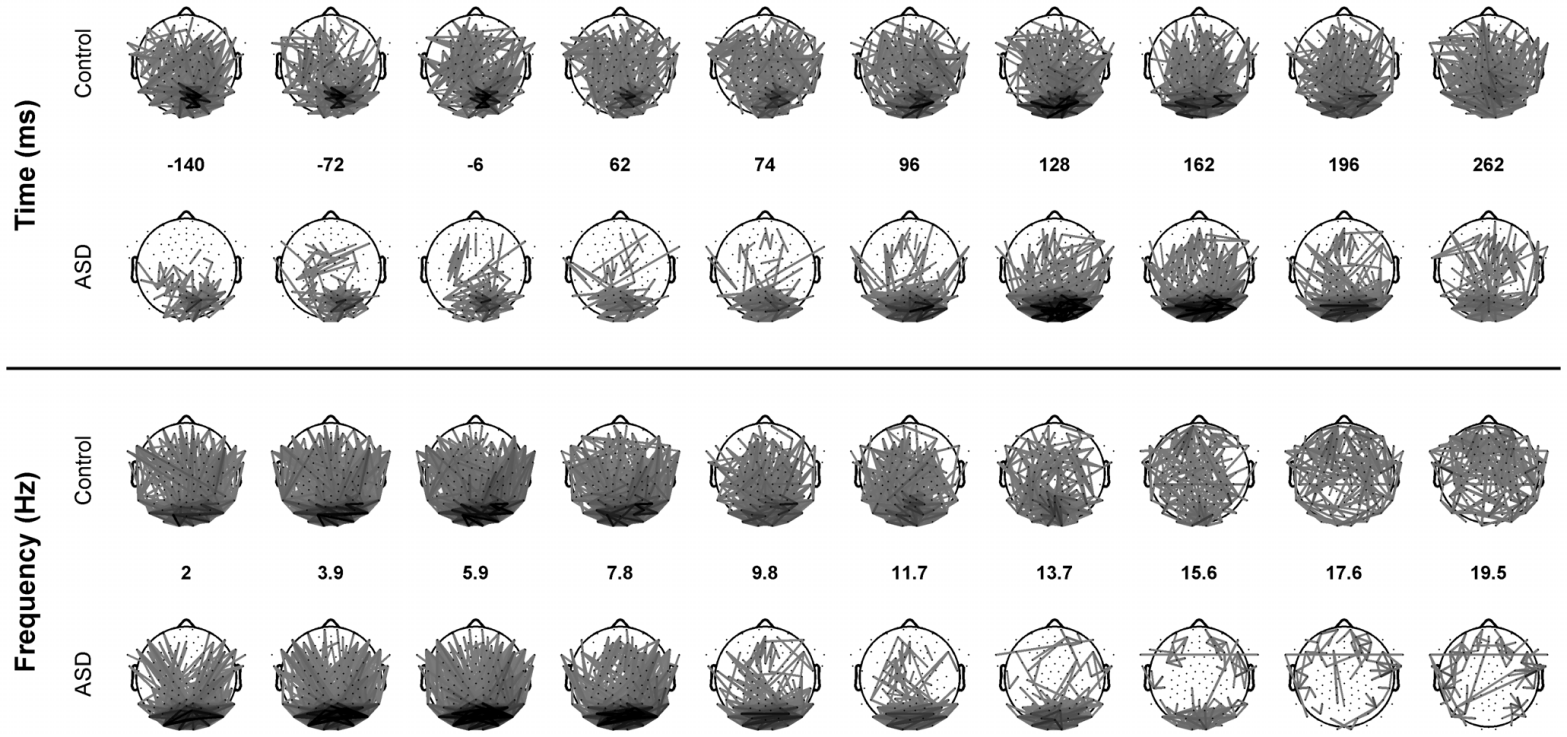
ICOH values are mapped to a line connecting the involved channels, the darker the line the higher the ICOH value. Frequencies and times are the same as in figure 4. For clarity, only channels whose ICOH are more than ten standard deviations than the control baseline are shown.

In order to offer a more detailed topographic view of the coherent activity and its relation to time and frequency, two different mappings are provided (Figures 3 and 4). In figure 3 averages by channels are shown. That is, the average of the ICOH values between each channel (pivot channel) to all the others is mapped to the corresponding electrode position for that pivot channel. The top panel displays the average head for each group under this specific mapping for different time points at a fixed frequency (9.8 Hz). In the bottom panel the frequency is then varied and the time is fixed (196ms), around the time group differences are bigger according to figure 2. Note that in figure 2 the areas that are more synchronized in the alpha band are occipital for both groups. However it appears that, for the ASD group, this event related synchronization is much stronger and also more widely spread in occipital areas. This spread is towards more lateral occipital sites, which might include fusiform face areas and superior temporal sulcus. In pre-stimulus time the Control group appears to have higher central occipital alpha synchronization compared to the ASD group.

Figure 4 offers a more common, although more confusing, representation of ICOH values over the same times and frequencies as in figure 3. In order to avoid too much clutter, only channels whose synchronization are 10 standard deviations higher than the baseline of the control are shown. It is important to note that although the ASD head seems to contain fewer ICOH lines above the chosen threshold, the average coherence is bigger in the ASD group Moreover the number of channels whose coherence is bigger in the ASD group is in the majority (between 53 to 58%). In the top plot it can be seen that in the alpha band the ASD group exhibits weak and mostly local synchrony in the pre-stimulus time.

This local synchrony in alpha is significantly enhanced after stimulus presentation with increased long-range connections towards more anterior areas. For the Control group no pattern is evident other than a lateral spreading of synchronization over occipital channels. It is also notable that for both groups the mostly local pre-stimulus activation over occipital channels is medial and slightly left. In the lower panel it can be observed that the pattern of synchronization in both groups at time 196ms is similar for delta and theta frequencies and becomes more long range and anterior for the Control group at alpha and Beta frequencies. These two mappings (figures 3 and 4) show an apparently different spatial organization of ICOH values during both the baseline and the post-stimulus period.

**Figure 5:**
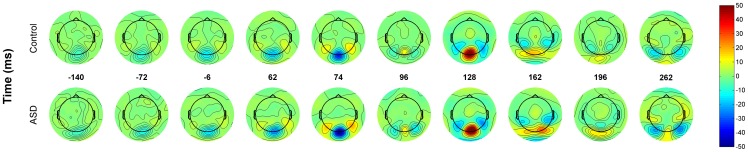
ERP averages across groups (Control and ASD) for each scalp site. Values are interpolated for areas between electrodes. Frequencies and times are the same as in figure 3.

**Figure 6:**
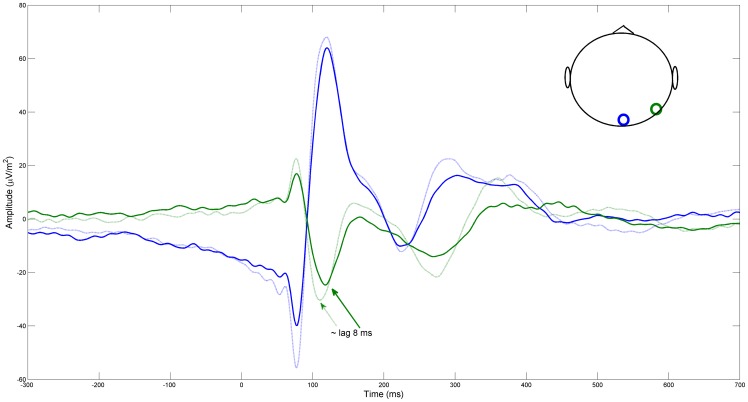
Raw ERP with all subjects pooled together. Green traces correspond to the occipital lateral location displayed with a green circle in the inset while blue correspond to the central occipital location. Solid line correspond to the Control group and the dashed one to the ASD group. The red arrows show an 8ms delay between the minimum of the solid line to the minimum of the dashed one.

Are these event-related imaginary coherency patterns correlated to event-related potentials (ERP) in the temporal and spatial domain? Figure 5 depicts the raw average EEG amplitude across groups for each scalp location for the same frequencies and windows as shown in figure 3. A direct comparison between figures 3 and 5 should be done cautiously since, in figure 3, the values that correspond to each channel are average values across all its connections. Figure 5 shows that the main source of activity is also located across the occipital area, however the differences between groups seem not to be as prominent as in figure 3. In figure 6 we present a closer look at the temporal course of two ERP traces generated at two occipital regions, central (blue) and lateral (green). Clearly, the period of maximum activity extends roughly from 70 to 400 ms. This corresponds to the period of the highest values of ICOH as depicted in figure 1.

Figure 6 also indicates that there is a first component peaking around 80ms, which seems to be in-phase (zero phase difference) for each group and in anti-phase (pi-phase difference) for different sites. This type of response, which seems to have the same phase profile for both sites, should correspond to a unique source, most likely C1, which is an early visual evoke response thought to be localized in Brodmann’s Area 19. From this moment, the ERPs of both groups seem to deviate in their phase course. In particular the green dashed line (ASD-Lateral) peaks around 8ms ahead of the same site in the Control group. Since there is also a non-zero lag respect to the peak at the other site in the same group, this could translate into an elevated ICOH value for some of the frequency components between these two areas. Also, since both groups show a different phase profile at this component, it is expected that their ICOH values should also differ for some frequencies when this lateral occipital electrode is compared to other electrodes. It is also noteworthy that the instantaneous frequency of the ERPs seems to move from higher to lower frequencies in time, concomitant with the pattern of maximal ICOH in the time-frequency plot of figure 1.

### Subject x COI x Time x Frequency

**Figure 7:**
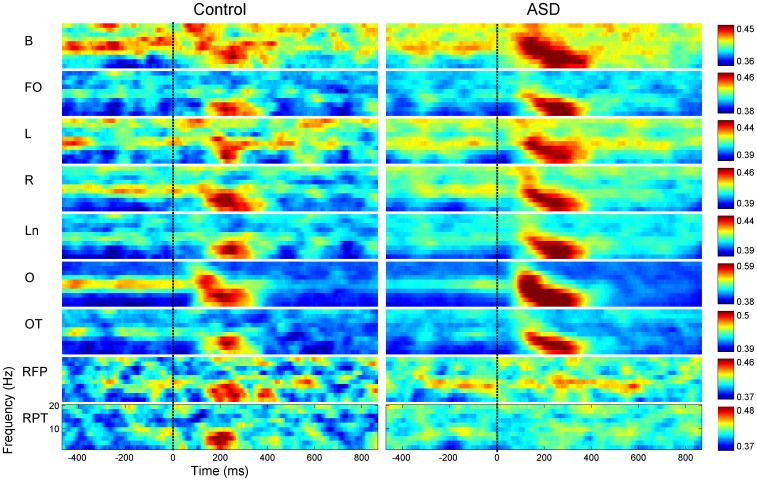
Time-frequency plots of ICOH for 9 different COI. Frequencies span only from 0 to 20: Control, right: ASD. Each row corresponds to a different COI, from top to bottom: bilateral (b), frontal-occipital (fo), left (l), right (r), ln (long), o (occipital), occipital temporal (ot), right frontal-parietal (rfp), right parietal-temporal (rpt). See each site description on table 1.

In order to facilitate the interpretation of the results by further aggregating information into selected types of connections, the 5995 connectivity values were averaged into 48 groups denoting Connections of Interest (COI) (Table 1). By corresponding the definitions of COI in the table, an ICOH value between two given electrodes can participate in the average of more than one COI. Broadly speaking, when collapsing across all other dimensions ICOH is more elevated across all frequencies for the ASD group. Differences in ICOH are notable around the post-stimulus period in most COI, particularly in occipital areas and in areas connected to occipital channels (figure 7). A decreased coherent activity during the baseline period relative to control is also detected for the occipital channels at alpha frequencies. This effect can also been seen in figure 3, top panel. Although most COI display an increased ICOH in the ASD group during the post-stimulus period some exceptions also occur, all of them in connections with parietal channels participation (see the panels labeled RFP and RPT).

**Table 1:** Connections of Interest (COI). Connectivity values (ICOH) are averaged into 48 groups of interest.

Abbreviation	Name	Description
b	Bilateral Symmetric	Only conn. between each channel and their equivalent channel on the contralateral side
bf	Bilateral Symmetric Frontal	Bilateral Symmetric ∩ Frontal
bo	Bilateral Symmetric Occipital	Bilateral Symmetric ∩ Occipital
bp	Bilateral Symmetric Parietal	Bilateral Symmetric ∩ Parieral
bt	Bilateral Symmetric Temporal	Bilateral Symmetric ∩ Temporal
lr	Bilateral	All contralateral connections (no necesarily symmetric)
ff	Bilateral Frontal	Bilateral ∩ Frontal
oo	Bilateral Occipital	Bilateral ∩ Occipital
pp	Bilateral Parietal	Bilateral ∩ Parietal
tt	Bilateral Temporal	Bilateral ∩ Temporal
f	Frontal	All conn. between Frontal channels
o	Occipital	All conn. between Occipital channels
p	Parietal	All conn. between Parietal channels
t	Temporal	All conn. between Temporal channels
l	Left	All conn. between left lobe channels
r	Right	All conn. between left right channels
lf	Left Frontal	Left ∩ Frontal
lo	Left Occipital	Left ∩ Occipital
lp	Left Parietal	Left ∩ Parietal
lt	Left Temporal	Left ∩ Temporal
rf	Right Frontal	Right ∩ Frontal
ro	Right Occipital	Right ∩ Occipital
rp	Right Parietal	Right ∩ Parietal
rt	Right Temporal	Right ∩ Temporal
sh	Short	All conn. between channels separated by less than 4 cm
ln	Long	All conn. between channels separated by more than 10 cm
ot	Occipital-Temporal	All conn between the mentioned areas on the same lobe
op	Occipital-Parietal	All conn between the mentioned areas on the same lobe
of	Occipital-Frontal	All conn between the mentioned areas on the same lobe
tp	Temporal-Parietal	All conn between the mentioned areas on the same lobe
tf	Temporal-Frontal	All conn between the mentioned areas on the same lobe
pf	Parietal-Frontal	All conn between the mentioned areas on the same lobe
lot	Left Occipital-Temporal	Left ∩ Occipital-Temporal
lop	Left Occipital-Parietal	Left ∩ Occipital-Parietal
lof	Left Occipital-Frontal	Left ∩ Occipital-Frontal
ltp	Left Temporal-Parietal	Left ∩ Temporal-Parietal
ltf	Left Temporal-Frontal	Left ∩ Temporal-Frontal
lpf	Left Parietal-Frontal	Left ∩ Parietal-Frontal
rot	Right Occipital-Temporal	Right ∩ Occipital-Temporal
rop	Right Occipital-Parietal	Right ∩ Occipital-Parietal
rof	Right Occipital-Frontal	Right ∩ Occipital-Frontal
rtp	Right Temporal-Parietal	Right ∩ Temporal-Parietal
rtf	Right Temporal-Frontal	Right ∩ Temporal-Frontal
rpf	Right Parietal-Frontal	Right ∩ Parietal-Frontal
rsh	Right Short	Right ∩ Short
lsh	Left Short	Left ∩ Short
rln	Right Long	Right ∩ Long
lln	Left Long	Left ∩ Long

Notation and definition of these groups in terms of sensor areas is shown in the table.

**Figure 8:**
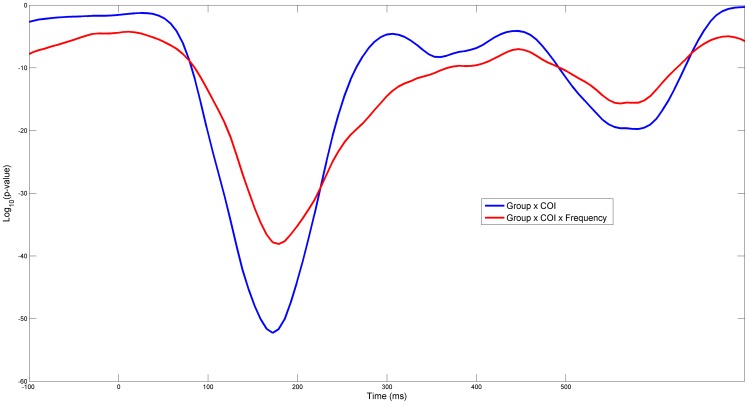
Curves are base-10 logarithms of Pvalues from a number of 3-way ANOVA’s. ANOVA’s were repeated for 181 sliding time windows. Within each single time window 20 ICOH values were averaged corresponding to a period of 128ms. The 3 factors in the analysis were, the two Groups, Frequencies (considered here as a continuous factor) and COI. Only interaction effects with Groups are shown.

As shown in figure 2, the terms in the anova analysis dropped their p-value around the post-stimulus period. In order to address statistically differences in spatial patterns, a second ANOVA was also carried on using the 48 regions of connectivity (COI) as a factor. Figure 8 shows the interaction effects of Groups and COI and the 3 factor interaction effect with Frequency. It can be clearly observed that even though these lines are always below the p = 0.05, COI interacts strongly with groups reaching a minimum at approximately 180ms. This may be a further indication of a different processing style for the ASD group.

**Figure 9:**
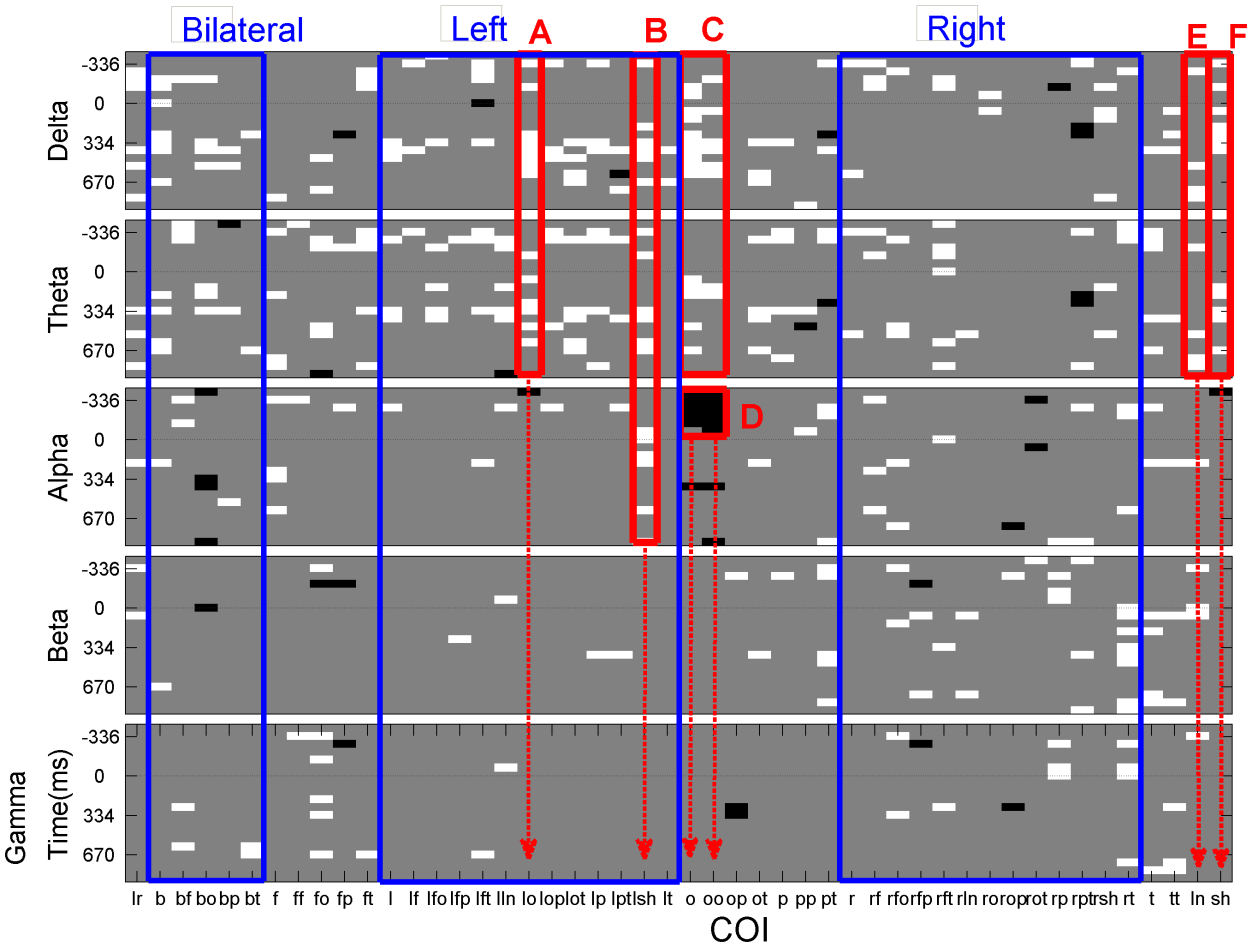
Results from a Mann–Whitney *U* test over 5 putative EEG frequency bands, 48 COI and 20 disjoint time windows of 64 ms each. There is one panel for each frequency band. Within each panel y-axis denotes time and x-axis COI. White corresponds to regions in which there is a significant difference between both groups where ASD is bigger in ICOH values than Control group. Black denotes significant differences where control is higher. Gray means there are not significant differences. Blue rectangles are shown only to organize the spatial information into 3 distinct anatomical, bilateral, left and right COI. Red rectangles are labeled with red uppercase letters and are used to highlight regions and frequencies that seem to contain a high frequency of significant values.

In figure 9 we present a graph derived from an analysis that more clearly summarizes the differences between both groups by frequency, time and COI. The data was averaged into 5 putative EEG frequency bands, the 48 COI and also 20 disjoint time windows of 64ms each. On this new set a total of 5*48*20  =  4800 Mann–Whitney *U* test were completed. In this figure white areas correspond to significant differences between groups where ICOH of ASD group is higher and black correspond to areas where ICOH of Control group is higher. Gray areas correspond to non-significant differences. As figure 9 shows, the black areas are relatively rare compared to the white ones. Some specific areas are emphasized in red and labeled for an easy reference.

We will now summarize some the most notable characteristics of this graph:

A: Increased left occipital ICOH for ASD for Delta and Theta  frequencies. In general ASD>Control seems to be particularly  true for Alpha and Theta bands over the left hemisphere.

B: ASD>Control over short connections over the left side for  Delta, Theta and Alpha.

C: ASD>Control over occipital connections for Delta and  Theta frequencies.

D: ASD<Control during the pre-stimulus time for occipital  channels. This is a strong effect that can be observed also in  figures 3 and 7.

E: ASD>Control for long connections for Delta and Theta  frequencies.

F: ASD>Control for short connections for Delta and Theta  frequencies.

Golland et al., (2008) [27] proposed a useful and flexible single test to address group differences based on a collection of features. The test is non-parametric and is based on classification performance. More specifically, the test is based on comparing the classification error for the original groups to the population of errors obtained by applying the same classifier to new groups resulting from a randomization of labels. A measure of accuracy and an F-score was obtained for each randomization of labels to account for classification performance. This randomization was carried out 2000 times. This test provides a measure of the statistical significance of the classifier performance as well as general group differences. In our study a feature vector for each subject is made using all frequencies averaged over 13 non-overlapping windows (2 frequencies in each), all COI and also all time points averaged in 4 non-overlapping windows. A total of 13*48*4  =  2,496 feature vector for each participant was fed into the classifier. A support vector machine with a linear kernel was used, and the classification error was determined by 200 rounds of crossvalidation. Since the number of members in each class is slightly unbalanced (31 vs 72), to avoid dropping information from the ASD class to balance the training sets, we applied the SMOTE algorithm [28] in which the minority set is complemented with synthetic feature vectors produced from the training data by interpolation over random vectors within each specific neighborhood. In each crossvalidation round both training sets had the same number of members.

As mentioned, the F-score, a measure that combines precision and recall, was used along with the classifier accuracy to measure the classifier performance. The original F-score was 0.88, which was bigger than 99.5% of the respective values from the randomized classes. The original accuracy was 0.80 bigger than the 99.5% of the respective values from the randomized classes. Both represent statistical significant values (alpha < 0.01), which confirms that the two classes are significantly different for the set of features chosen.

## Discussion

The analysis we have presented here presents an account of differences in cortical synchronization patterns in the autistic population via a methodology that offers a credible perspective on functional connectivity, although not necessarily a complete one. We have presented evidence of solid group differences which provide support for the adequacy of this measure, in particular, differences associated with the post-stimulus period. These results challenge the idea of functional under-connectivity in individuals with autism. We also showed that relatively high classification accuracies can be obtained from measures of the imaginary part of coherence alone.

By looking through the lens of a functional connectivity tool, the Imaginary Part of Coherency, we presented clear differences between the brains of autistic and control children at a wide range of frequencies, locations and times. This measure avoids volume conduction effects, which are instantaneous and which greatly affect the traditional coherence analysis, by focusing on the synchronized activity that occurs at a certain delay. The method is essentially artifact-free, that is, significant differences in activity are real and cannot be produced by artifacts [18], However the method will fail to see any real synchronized activity occurring instantaneously (at phase 0°).

It is also important to keep in mind, when comparing our results to others on scalp EEG, that the specific montage in which the data was studied can greatly misrepresent values of coherency/synchronization [16], [23]. Well-designed experiments and potentially excellent data can be easily destroyed by failing to modify the original referential montage before the functional connectivity analysis is carried out. We took special care in transforming the original referential montage to a reference-free one in which the phase of each channel is not distorted by the phase and amplitude of the reference channel. Having overcome these technical difficulties, we captured an interesting transient spatio-temporal structure, which agrees with previous studies on face and emotion processing in many aspects, offering some extra validation to the relevance of the methodology. In particular, the specific pattern we have described of bigger ICOH values for different latencies at different frequency bands, where latencies increase moving from alpha to lower frequencies (figure 1 and 7), has been reported before [29], [30] in the context of Event Related Desynchronization due to the emotional content of a face. In these previous studies researchers also noted the role of the delta band in recognizing the emotional content of the face. This peaks around 320ms and continues for a few hundred milliseconds, while an earlier component related to an arousing facial stimuli (not neutral) peaks around 200ms. That is, these peak modulations were attributed respectively to the emotional discrimination and to the attentional significance of face. These events coincide with the local minima in the p-value curve for Task and Group in figure 2 as well as the local minima of the interaction Group x Task. Another interesting feature, the wide area of synchronization over occipital channels in figure 3 towards the left and right (but predominantly right), may be produced by abnormal synchronization of the fusiform face area and Superior Temporal Sulcus, see also the right dominance to face perception [29]–[31].

The ANOVA analyses (figure 2 and 8) were carried over a sliding window to show its profile in the time domain and the particular timing at which it drops significantly. In both cases, based on the interaction terms with Groups, this analysis seems to show a different mechanism of processing the stimulus. While some initial studies reported a weak or non-existent activation in the hemodynamic response in areas associated to face perception, including the amygdala [32], [33] a latter study [31] found no such deficit in activity when the eye fixation was controlled. In another study it was found that the activation in these areas was strongly and positively correlated with the time the autistic group spent fixating the eyes [34]. Since the ASD group has typically diminished gaze fixation in relation to the control group, this explains the presumed lower activation reported before. In our setting, eye fixation is monitored and only trials whose gaze has been maintained for more than 100ms on the face were accepted for further processing. Thus, we should not expect a reduced activity in these areas associated with face processing. Moreover we found a generally increased connectivity in most areas, with some exceptions in connection to parietal channels.

As with any other result derived from measures of connectivity, ours have to be interpreted carefully and critically. Similar to the term “complexity,” as applied to brain dynamics, “functional connectivity” has many meanings according to the specific mathematical formulation. In fact most of these formulations incorporate artifacts thereby capturing more than the intended purpose, and some produce only partial accomplishment by failing to capture some real connectivity. Therefore, results derived from different recording modalities and mathematical methods for connectivity are not directly comparable. For example, when an fMRI study claims poor synchronization, as in [4], it may refer to correlation between voxels or entire ROI’s. Consider that while the correlation between two signals dominated by a single frequency is maximal when there is a shift of phase zero or π between them, the ICOH would have the opposite behavior, minimal at those values and maximal at π/2 or 3π/2. Therefore, we are not in a position to claim we have completely disproven any hypothesis formulated in terms of “functional connectivity” as a physiologist would understand it, nor can we make claims of anatomical nature derived from our findings. However we can positively say that there are notable differences in functional connectivity patterns, specifically, that functional connectivity that occurs at some non-vanishing time lags. Since non-interacting sources cannot produce a non-vanishing imaginary part, they cannot produce significant differences between groups other than by chance. The ANOVA’s we have presented, the resampling test for classification accuracies and the Mann–Whitney *U* test analysis by brain areas and frequency bands, all produce different confirmation of these notable differences.

In general we found increased ICOH in the ASD group compared to controls (see the relative larger white area in figure 9 in comparison to the black one for an easy confirmation of this). This seems to be in disagreement with the most accepted theory of underconnectivity in autism, which, in some results, tends to associate structural to functional connectivity or, more importantly, a lack of integration of information between specialized areas, with a decrease in functional connectivity. We think, however, in terms of metastability [11], where more essential, for proper information processing, is the flexibility in forming and dissolving synchronized activity among different cell populations. Since there are both excitatory and inhibitory connections a decrease (or increase) in physical connectivity per se does not guarantee a given tendency towards synchronization. The balance between inhibition and excitation and other network parameters is required to affect network dynamics toward synchronization. More in agreement with our findings is [35], where it is proposed that the balance between excitation/inhibition leans in favor of excitation, which accounts for the relatively large proportion of seizure and spike activity documented in the brains of children with ASD. A large proportion of significant values for short connections at theta and delta frequencies is evident in In figure 9 (F). This agrees with most studies that find enhanced local synchronization in ASD. However we failed to see decreased bilateral (9 second column) and long range synchronization 9 (E).

Recent attempts have been made towards finding biomarkers for autism in EEG [5], [36], [37]. Tsiaras et al. [36] present an interesting solution to managing the cumbersome information derived from connectivity analysis by collapsing information in a number of graph connectivity parameters. These parameters, derived from three different connectivity measures, were used as features in building a biomarker. Bosl et al. [37] used Modified Multiscale Entropy, a statistic that is computed for each single channel, giving some measure of the complexity of the time course. Although both approaches are interesting, the classes used for testing and training the classifiers are rather small, with not much room left for a validation of the accuracies presented. Further, while the accuracies reported result from valid rounds of crossvalidation, the fact that many tests are presented in these papers, would definitely contribute to increase the accuracies of the setting or group with the best performance [38]. A more recent result [5] reports a very high classification accuracy on a large number of ASD and Control cases. In particular for the group ages 2 to 4, which is comparable to our range, the Control group contained 85 subjects while the ASD group 216. Using the traditional coherence measure on a segment of spontaneous EEG, the reported total accuracy was bigger than 97%. This impressive performance was achieved by using the traditional coherence analysis on 24 channels and 16 frequency bands in the 1–32 Hz range. The number of variables used in this classification was reduced by applying PCA to the original set of 4416 variables. Interestingly, a single coherence value per pair of channels and frequency was calculated for each subject by using a time window 2 seconds wide over a segment 8 to 20 minutes long. Taking into account the aforementioned limitations of the coherence measure, plus the fact that the EEG is highly non-stationary over these scales (8–20min), the analysis produced impressive results. These facts could lead to further progress through research that investigates why these brains can be differentiated on averages over such relatively large scales and why volume conduction seems to sharpen rather than attenuate group differences. Are these high accuracies resulting from real processes of functional connectivity only? Even if they are not, there is no doubt from a practical standpoint the authors have produced a very useful and simple biomarker for the autism phenotype in childhood.

In our study, in comparison to Tsiaras et al. [36] and Bosl et al. [37], the classification accuracy of 80% seems already good enough for a single attempt with a vector containing all features. The result could be improved, through careful selection of features but in the absence of a third hold-out final validation set, it would not be legitimate to do so. We did not follow this approach since we considered that there were not enough data left for a solid performance of the classifier. On the other hand we believe that the findings of Duffy & Als [5], based on spontaneous, 24-channel EEG, offer a more practical solution to the biomarker problem. However, its relevance should be further investigated from a physiological standpoint by comparing it to results like ours which are theoretically more reliable measures of functional connectivity. Finding a reliable biomarker for ASD in a relatively inexpensive recording modality such as scalp EEG will greatly help in the timely diagnosis of this syndrome and may also enhance the ability to test the effectiveness of different treatments.

Since only a few studies on autism have, so far, used measures of functional connectivity from scalp EEG/MEG, our results should provide further motivation to look more deeply into the possibility of different processing styles from the perspective of electrophysiological recordings, since this approach appears to be very well suited to capturing fast transient activity, using different experimental cognitive paradigms. A similar idea has recently been proposed to use MEG to study executive functions [8].
